# Real-Time Duplex Applications of Loop-Mediated AMPlification (LAMP) by Assimilating Probes

**DOI:** 10.3390/ijms16034786

**Published:** 2015-03-03

**Authors:** Ryo Kubota, Daniel M. Jenkins

**Affiliations:** 1 Diagenetix, Inc., Honolulu, HI 96822, USA; 2 Department of Molecular Biosciences and Bioengineering, University of Hawai'.i, Mānoa, 1955 East-West Road, Honolulu, HI 96822, USA; E-Mail: danielje@hawaii.edu

**Keywords:** molecular diagnostics, isothermal nucleic acid amplification, *Ralstonia solanacearum*, Race 3 Biovar 2, *Salmonella enterica*, point-of-care testing, on-site detection, on-site diagnostics

## Abstract

Isothermal nucleic-acid amplification methods such as Loop-Mediated isothermal AMPlification (LAMP) are increasingly appealing alternatives to PCR for use in portable diagnostic system due to the low cost, weight, and power requirements of the instrumentation. As such, interest in developing new probes and other functionality based on the LAMP reaction has been intense. Here, we report on the development of duplexed LAMP assays for pathogen detection using spectrally unique Assimilating Probes. As proof of principle, we used a reaction for *Salmonella enterica* as a model coupled with a reaction for λ-phage DNA as an internal control, as well as a duplexed assay to sub-type specific quarantine strains of the bacterial wilt pathogen *Ralstonia solanacearum*. Detection limits for bacterial DNA analyzed in individual reactions was less than 100 genomic equivalents in all cases, and increased by one to two orders of magnitude when reactions were coupled in duplexed formats. Even so, due to the more robust activity of newly available strand-displacing polymerases, the duplexed assays reported here were more powerful than analogous individual reactions reported only a few years ago, and represent a significant advance for incorporation of internal controls to validate assay results in the field.

## 1. Introduction

Isothermal nucleic acid amplification technologies have a significant advantage over polymerase chain reaction (PCR) as they can be implemented in a single step process at a constant temperature [1]. Removing the constraint for rapid thermal cycling enables diagnostics to be conducted in small, simple, and low-power instruments in comparatively primitive conditions [2,3,4,5,6]. These advantages have also lead to considerable interest in using these techniques to conduct molecular diagnostics at the site of infection or contamination, *i.e.*, point-of-care testing [7,8]. Therefore, a variety of isothermal nucleic acid amplification technologies have been developed over a decade [1,6]. Most of these technologies require molecular machines and potentially unstable cofactors/coenzymes in addition to DNA polymerase to successfully replicate DNA. For example, Helicase-dependent amplification (HDA) utilizes DNA helicase to denature double stranded DNA (dsDNA) into single-stranded template for primers to anneal to and initiate the amplification process [9]; Nicking Enzyme Amplification Reaction (NEAR) utilizes nicking enzyme to introduce nicks into dsDNA from which polymerization can originate; Recombinase, Polymerase Amplification (RPA) utilizes recombinase which forms complexes with the primers to facilitate the annealing of primers into a double stranded template [10,11], and; Nucleic Acid Sequence Based Amplification (NASBA) mimics the retrovirus RNA replication system with RNase H and reverse transcriptase [12]. In comparison, Loop-mediated isothermal AMPlification (LAMP) is an especially promising technology in that it requires only a single enzyme (strand displacing polymerase) to amplify DNA in a truly single step reaction [13]. LAMP uses specially designed primers which are able to anneal to destabilized segments of the DNA template at optimal reaction temperatures around 65 °C [14]. In addition to four essential LAMP primers (F3, B3, FIP, and BIP), loop primer(s) can be designed which result in a more intense and accelerated reaction [15] that is more selective to the target sequence, evidently by facilitating the annealing of inner primers [16]. One of the unique characteristics of the LAMP reaction is the prolific generation of insoluble Magnesium Pyrophoshpate as a byproduct of the intense polymerization reaction, which can allow end-point classification of reactions by direct visual observation of turbidity [17], or real-time classification using turbidimetric measurements [18]. Since the same process also results in a decrease of Magnesium ion concentrations as the LAMP reaction proceeds, metal-chelating fluorescent indicators such as calcein with MnCl_2_ [19] and hydroxyl naphthol blue (HNB) [20] can be used to generate a spectrally selective signal that is a more reliable indicator than turbidity [21].

Since the LAMP reaction generates a prolific number of self-replicating amplicons which can each initiate a subsequent LAMP reaction, practical application of LAMP requires fastidious preparation of reaction mixes in facilities where contaminating DNA is not present, and in any circumstance it is ill-advised to open completed LAMP reaction tubes for subsequent analysis of amplicons. We previously developed Assimilating Probes to allow sequence-specific real-time monitoring of LAMP reactions directly in the reaction tube without subsequent molecular analysis [16]. This technology allows one-step application of LAMP with higher specificity, and significantly lowers the risk of contaminating subsequent reactions. The reaction can be monitored in real-time with an inexpensive handheld device, leveraging the simple LAMP process for mobile diagnostics and point-of-care testing [3].

To succeed commercially especially for distributed point-of-care applications, diagnostic technologies must be simple to use, rapidly generate an unambiguous signal, and include internal controls to validate the outcome. Lateral-flow devices (LFDs) are excellent examples of technologies meeting these criteria. These devices consist of a strip across which the sample flows by capillary force, and interaction of specific biological markers with bioaffinity probes immobilized on the strips results in appearance of a clearly visible bands. Typically a control band is included to ensure that the reagents are still functional. Most commonly the recognition is based on immunological interactions of antibodies with a specific antigen. However, development of highly specific antibodies is a time-consuming, labor-intensive, and expensive process that is especially difficult for unculturable pathogens, or for discriminating specific isolates of pathogens that do not display clearly differentiated surface antigens. In contrast, new tools for manipulating, synthesizing, sequencing, and analyzing nucleic acids can result in rapid identification of highly specific gene sequences of interest for diagnostic applications. Implementing nucleic acid hybridization with the LFD format, often referred to as nucleic acid lateral-flow (NALF), can be an effective method of leveraging the simplicity of LFD and the power of gene-based diagnostics [22]. This approach has been demonstrated for example to detect amplicons from specific LAMP assays [23]. As described above, one compelling advantage of LFDs is that they can contain an internal control marker to provide validation of the assay results. Similarly, nucleic acid amplification such as with LAMP involves complex biochemical interactions which may fail due to inhibition or loss of activity of the different reaction components, so that incorporation of proper controls to validate assay performance is critical. This is especially true when analyzing complex environmental, food, or clinical samples with unpredictable composition and numerous potential inhibitors. Tomlinson *et al.* successfully demonstrated multiplexed LAMP (mLAMP) analyzed with LFD for detecting the fungal pathogen *Phytophthora ramorum* with an internal control [24]. The LAMP assay contained primers to amplify DNA from the pathogen, as well as additional primers to amplify DNA from the host plant to confirm the adequacy of sample preparation and activity of the test reagents. Several other approaches for analyzing mLAMP reactions have also been reported but these generally require additional analyses such as restriction enzyme digestion to differentiate the amplicons, thereby failing to capitalize on the speed and simplicity of LAMP technology [25,26,27]. Real-time mLAMP has also been reported using “release” of quenching (DARQ) technology [28] with a probe architecture identical to Assimilating Probes [16]. In this implementation, spectrally unique fluorescent probes for different LAMP amplicons enabled simultaneous monitoring of duple LAMP reactions in real-time, obviating the need for end point analysis using LFDs or other approaches.

Here we report real-time duplexed LAMP application using Assimilating Probes to include internal controls or to enable specific pathogen sub-typing. Specifically we demonstrate duplexed LAMP for the detection of *Salmonella enterica* subsp. *enterica* ser. Typhimurium [3], integrated with an internal control reaction based on a previously published primer set for *enterobacteria phage* λ [15]. To demonstrate pathogen sub-typing capability in a duplexed format, we also applied duplexed LAMP for the bacterial wilt pathogen *Ralstonia solanacearum* (Rs) [29,30] and specific sub-populations of the same pathogen commonly designated as Race 3 Biovar 2 (R3B2) [29,31,32]. While Rs affects a wide variety of important crops and is extremely persistent in warm, humid tropics, R3B2 strains of Rs are of special concern in the US because they are adapted to cooler climates and could result in serious economic impacts to agricultural production in North America if they become established there [29].

## 2. Results and Discussion

### 2.1. Individual LAMP (Loop-Mediated Isothermal AMPlification) Reaction Results

Candidate Rs species level LAMP primer sets were validated by following previously described steps [33] using a total of 268 bacterial isolates including 264 Rs-complex strains and four non Rs-bacterium. The primer set egl62 successfully amplified DNA from 264 Rs-complex strains and not DNA from four non Rs-bacterium. Then, Assimilating Probes were designed based on the previous report [17] and reassessed the specificity using the same bacterial isolates. Detection limits for individual (singleplexed) LAMP reactions conducted under the conditions described here resulted in detection limits (*i.e.*, consistent detection of triplicate reactions) of 500 fg of DNA for reactions with *Salmonella enterica* (Se), Rs, and Rs R3B2, equivalent to fewer than 100 genome equivalents per reaction in each of these cases (Table 1). The detection limit for the singleplexed *phage* λ reaction was only 50 fg, but this corresponded to a significantly larger number of template DNA copies (1000; Table 1) of the comparatively small phage genome. This result was somewhat surprising given our expectation that a smaller and putatively less complex DNA structure would be more reactive, and suggests that the phage DNA may have been subjected to greater degrees of digestion/decomposition prior to analysis, or that the greater entropy of smaller DNA molecules makes the initiation of the LAMP process more thermodynamically unfavorable. The detection limit for the singleplexed reaction for Rs R3B2 DNA was consistent with previously reported values under the same conditions [16], but the detection limit for Se DNA was significantly lower under the conditions reported here compared to the value (1.5 × 10^4^ genomic equivalents) reported for reactions with the same primer set but a different polymerase (*Bst* DNA polymerase, New England Biolabs, Ipswich, MA, USA) [3]. This significant discrepancy in detection limits is largely attributable to the more robust enzyme and optimized master mix used in this report, though it is noteworthy that other strand displacing enzymes with improved activity, thermal stability, and other characteristics have been reported [34,35] and are commercially available.

### 2.2. LAMP with Internal Control: Salmonela Enterica and Phage λ Detection

**Figure 1:**
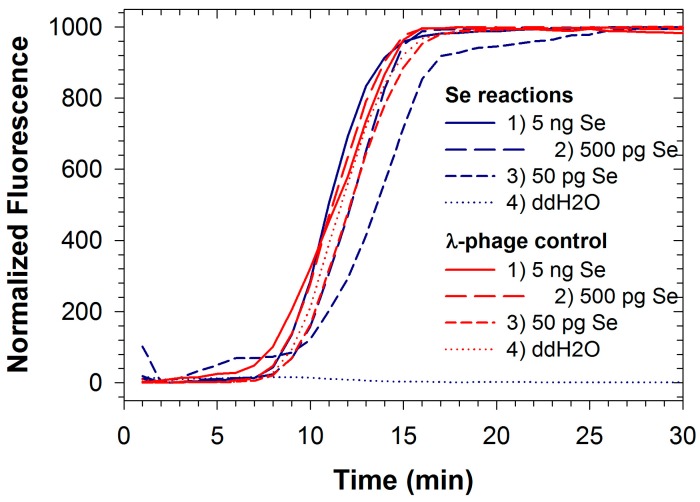
Duplexed LAMP reactions for *Salmonella enterica* (Se) DNA (blue lines) run with internal control reaction for *enterobacteria phage* λ (red lines).

Fluorescence signals for both FAM and TAMRA from Se/λ-phage were recorded simultaneously during duplexed LAMP reactions (Figure 1). Analogously to quantitative PCR, reactions containing smaller concentrations of *Salmonella* DNA resulted in delays in the threshold time. However threshold times for the control reaction with λ-phage DNA remained roughly constant as template DNA was standardized for these reactions. The detection limit for *Salmonella* DNA using the duplexed reaction was about 50 pg (9.8 × 10^3^ genomic equivalents; Table 1), two orders of magnitude greater than the detection limit for the singleplexed reaction, and similar in magnitude to the singleplex detection limit for the reaction when using *Bst* polymerase [3]. This indicates that competitive effects for available nucleotides and polymerase occur when including additional primer sets and template DNA in the reaction. The development of efficient multiplex PCR requires rigorous optimization because numerous factors can influence the sensitivity and specificity, including the relative concentration of primers, buffer, magnesium chloride, dNTPs, annealing temperatures, and other reaction components/conditions [36].

**Table 1:** Effective doubling times for different reactions and reaction conditions.

Reaction	Single				Duplex					
Template DNA	*Salmonella*	*phage* λ	Rs R3B2	Rs R3B2	*Salmonella* and *phage* λ		Rs R3B2 (UW551)		Rs (GMI1000)	
Primer Set	Se	λ-phage	egl62	rk2208.1	Se and λ-phage		egl62 and rk2208.1		egl62 and rk2208.1	
Probes	Se	λ	egl62	rk2208.1	Se	λ	egl62	rk2208.1	egl62	rk2208.1
τ_D_^a^	28.1 ± 2.9	29.2 ± 1.6	47.6 ± 7.6	28.9 ± 3.4	39.1 ± 6.7	-	31.9 ± 3.4	37.9 ± 2.1	36.1 ± 3.1	-
LOD ^b^	98	1000	86	82	9800	-	860	820	860	-

^a^ Doubling time for given LAMP reaction (in seconds). ^b^ Detection limit (in genomic equivalents of template DNA per reaction).

Similar optimization processes are also required for multiplex LAMP reaction, and may be more challenging due to the tendency for LAMP reactions to run extremely prolifically (*i.e.*, there is a greater tendency for more favorable reactions to overwhelm less favorable ones). Further optimizations for duplexing *Salmonella* and *phage* λ reactions may result in improved detection limit for Se.

Competitive effects can also be inferred from the doubling times estimated for the Se reaction individually (28.1 s; Table 1) and when duplexed with the λ-phage reaction (39.1 s; Table 1). Both values are significantly lower than the doubling time (55.6 s) inferred from earlier reports of the individual Se reaction when using Bst polymerase [3], again illustrating the relatively robust activity of the polymerase used in this study. Interestingly, there are tradeoffs between detection limit and the resolution for quantitation resulting from differences in enzyme activity, as more robust enzymes result in observable amplification at lower pathogen titers but replicate amplicons faster so that quantitation based on threshold time results in diminished resolution. Under the conditions reported here, however, doubling times were still on the order of tens of seconds, so that improved resolution can largely be achieved simply by recording fluorescence values more frequently even for the fastest reactions reported here.

### 2.3. Duplex LAMP: Simultaneous Detection of Rs and Rs R3B2

**Figure 2:**
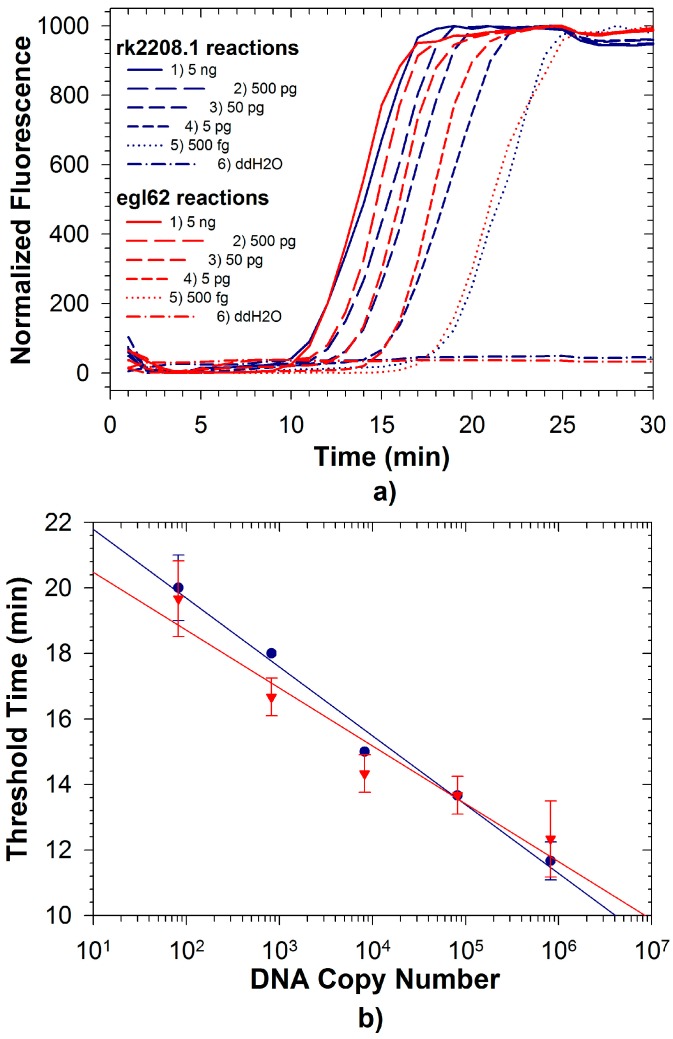
(**a**) Duplexed LAMP reactions with Rs R3B2-specific primer set rk2208.1 (blue lines) and Rs species-level primer set egl62 (red lines) conducted with template DNA from Rs R3B2 strain UW551; (**b**) Quantitative relationship of observed threshold times for rk2208.1 probes (•) and egl62 probes (▼) with UW551 DNA in duplexed reaction.

**Figure 3:**
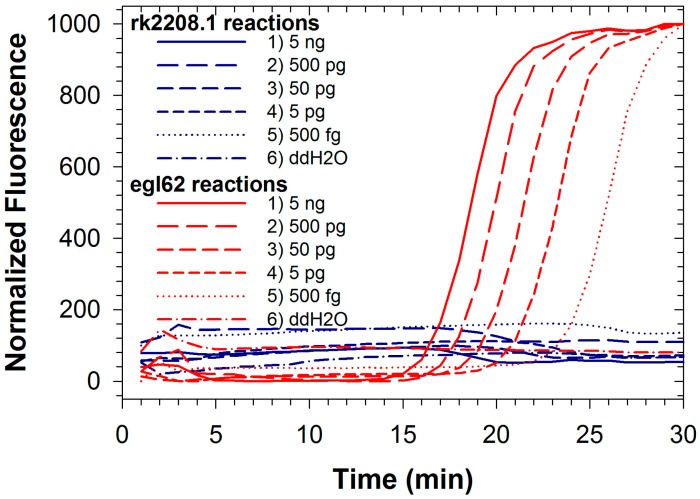
Duplexed LAMP reactions with Rs R3B2-specific rk2208.1 primer set (blue lines) and species-level egl62 primer set (red lines) conducted with template DNA from Rs strain GMI1000, which is not classified as R3B2.

Fluorescence signals for both FAM and TAMRA from Rs/Rs R3B2 were recorded simultaneously during duplexed LAMP reactions using Rs R3B2 (UW551) genomic DNA (Figure 2a,b). Detection limits for both reactions in the duplexed format described here were approximately 500 fg (less than 1000 genomic equivalents; Table 1), significantly better than the duplexed reaction for Se with an internal control. The detection limit for the egl62 primer set was identical whether testing the R3B2 strain of Rs, or the non-R3B2 strain, and as expected no amplification was observed with the rk2208.1 primer set when the non-R3B2 strain was tested (Figure 3; amplification from only egl62 primer set observed). The ability to identify R3B2 strains in a population of Rs is extremely important, as Rs is widely distributed in the tropics and commonly found on plant materials imported to the US, but only the R3B2 strains for which there are no other specific rapid tests are of concern to agricultural producers. Typically, plant materials imported into the US are tested for Rs using commercially available immunodiagnostic LFDs, which cannot discriminate the Select Agent R3B2 strains from other more ubiquitous strains. Therefore, all materials testing positive for Rs at ports of entry must be treated as being contaminated with a Select Agent until conclusively demonstrated otherwise using more selective tests [32]. The duplexed LAMP assay described here can be a valuable diagnostic tool to rapidly determine if a contaminated sample is infected with Select Agent R3B2 strains, while confirming detection Rs in the sample as an internal control reaction. We have already demonstrated the efficacy of the rk2208.1 LAMP primer set for direct detection of R3B2 strains on infected plant samples in the field [37]. The detection limit of the rk2208.1 LAMP primer set in the duplexed format observed in this study was an order of magnitude better than that of the same primer set previously reported [33] in a single reaction format using the less robust *Bst* polymerase. This suggests that improved performance of newer polymerases can more than compensate for competitive effects in a duplexed assay.

## 3. Experimental Section

### 3.1. Preparation of DNA Standards

*S. enterica* subsp. *enterica* ser. Typhimurium (ATCC #14028) was grown on Brain Heart Infusion (BHI) agar (Catalog No. 221610, Becton Dickinson, Franklin Lakes, NJ, USA) and incubated for 24 h at 35 °C. Rs strains (GMI1000 and UW551) were grown on modified tetrazolium chloride (TZC) agar medium [38] and incubated for 48 h at 28 °C. DNA was purified from all cultured cells with the Wizard Genomic DNA Purification Kit (Promega Corp., Fitchburg, WI, USA) according to the manufacturer’s instructions. DNA concentrations were quantified photometrically (absorbance measurements at 260 and 280 nm with an ND-1000 spectrophotometer, NanoDrop Technologies, Inc., Rockland, DE, USA). The copy number of template genomic DNA was estimated on a mass basis assuming genome sizes of approximately: 4.95 Mb with 53% GC contenet (*S. enterica* ATCC#14028) [39]; 5.8 Mb with 67.0% GC content (Rs strain GMI1000) [40]; 5.93 Mb with 64.5% GC content (Rs R3B2 strain UW551) [41], and; 48.5 kb for commercially available preparations of purified *enterobacteria phage* λ DNA (vendor item description; Catalog No. N3011S, New England Biolabs, Berkeley, MA, USA).

### 3.2. LAMP Primer Design

Primer sets used for *Salmonella enterica* [3], *enterobacteria phage* λ [15], and Rs R3B2 [33] are described in published research results. The sequence of the *egl* (endoglucanase) gene, which is regarded as a conserved virulence factor among Rs species [42], was used as the basis for designing a LAMP primer set selective to all isolates belonging to the Rs species. Publically accessible LAMP primer design software (PrimerExplorer, Eiken Chemicals, Tokyo, Japan, http://primerexplorer.jp/e/) was used to identify candidate Rs primer sets targeting the selected gene. Candidate Rs species level LAMP primer sets were screened against the same bacterial library as was used previously for validating the rk2208.1 primer set selective for R3B2 strains [33]. This library included a total of 268 bacterial isolates including 264 geographically diverse Rs-complex strains and four non Rs-bacterium. Detailed information on origin and biological characteristics of these isolates is available in the previous report [33].

### 3.3. LAMP Reaction and Assimilating Probes

LAMP primers and Assimilating Probes (Table 2) designed to selectively amplify and detect DNA from *Salmonella enterica*, *enterobacteria phage* λ, Rs R3B2, and Rs were synthesized by Integrated DNA Technologies (Coralville, IA, USA). LAMP reactions were performed in 25 μL (total volume) reaction mixtures containing 1.6 μM FIP and BIP, 0.2 μM of the F3 and B3 primers, 0.8 μM of the loop (F or B) primers, and template DNA obtained as described above. Primers were prepared in a commercially available Isothermal Master Mix without intercalating dye (Catalog No. ISO001-nd, Optigene, Inc., Horsham, UK) according to the vendor’s instructions. The enzyme used in Isothermal Master Mix is GspSSD DNA polymerase, large fragment, which has strand displacement activity and also reverse transcriptase activity. For all singleplex reactions, a total of 0.08 μM of each Assimilating Probe F strand, and 0.12 μM of the Assimilating Probe Quench strand were used. For the *Salmonella enterica* with internal control duplexed reaction, 0.04 μM each of Assimilating Probe strands Se F strand loopF and loopB, and 0.08 μM of λ-phage F strand loopF were combined with 0.2 μM of the Assimilating Probe Quench strand. Purified *Salmonella enterica* DNA were diluted to desired concentrations in ddH_2_O, and reaction mixes for *Salmonella* were prepared to contain 5 ng of commercially available *enterobacteria phage* λ DNA in each reaction as an internal control. For simultaneous typing of Rs and Rs R3B2, 0.08 μM each of the corresponding fluorescent strands (egl62 F strand loopF and rk2208.1 F strand loopB) of the Assimilating Probes and 0.2 μM of the conserved Assimilating Probe Quench strand were used. The template DNAs of Rs (strain GMI1000) and Rs R3B2 (strain UW551) cultures were diluted to desired concentrations in ddH_2_O. All reactions were carried out in capped 0.2 mL microtubes (Catalog No. 93001-118, VWR International LLC., Radnor, PA, USA) with temperature controlled in the block of a commercial real-time PCR instrument (iQ5 Real-Time PCR Detection System, Bio-Rad Laboratories, Inc., Hercules, CA, USA), at 65 °C for 30 min. Reactions were terminated by heating to 80 °C for 2 min. Real-time fluorescence values of on-going reactions with Assimilating Probes were measured every 1 min during the 30 min reactions. Assimilating Probes with FAM were monitored using filter position 2, and those with TAMRA were monitored using filter position 4. The “threshold time” t_T_ was estimated as the amount of time required for the fluorescence value to exceed a threshold value equivalent to the pooled average plus three standard deviations of the fluorescence values observed throughout the durations of triplicate negative control reactions [16].

**Table 2:** Loop-mediated isothermal AMPlification (LAMP) primer and Assimilating Probe sequences.

-	Nucleotide Sequence (5'→3')
**Se primer set**: designed to detect *Salmonella enterica*	
Se F3	GGCGA TATTG GTGTT TATGG GG
Se B3	TGAAC CTTTG GTAAT AACGA TAAAC TG
Se FIP ^1^	CTGGT ACTGA TCGAT AATGC CAAGT TTTTC AACGT TTCCT GCGG
Se BIP ^1^	GATGC CGGTG AAATT ATCGC ACAAA ACCCA CCGCC AGG
Se loopF	GACGA AAGAG CGTGG TAATT AAC
Se loopB	GGGCA ATTCG TTATT GGCG
**λ primer set**: designed to detect *Enterobacterio phage* λ	
λ-phage F3	GGCTT GGCTC TGCTA ACACG TT
λ-phage B3	GGACG TTTGT AATGT CCGCT CC
λ-phage FIP ^1^	CAGCC AGCCG CAGCA CGTTC GCTCA TAGGA GATAT GGTAG AGCCG C
λ-phage BIP ^1^	GAGAG AATTT GTACC ACCTC CCACC GGGCA CATAG CAGTC CTAGG GACAG T
λ-phage loopF	CTGCA TACGA CGTGT CT
λ-phage loopB	ACCAT CTATG ACTGT ACGCC
**egl62 primer set**: designed to detect *Ralstonia solanacearum*	
egl62 F3	CTGGA ACCAG AACTG GTACG
egl62 B3	ATAGC CGTTG CTGCG C
egl62 FIP ^1^	TGGTG CACCT CGAAG ACGAG GTCCG AACGG CACCG TCATG
egl62 BIP ^1^	CGATT CGTCC GGCCA GTCGC CAGTT GGTGA AGTCC TGC
egl62 loopF	CCGGG TCATT GATGC CCTT
**rk2208.1 primer set**: designed to detect *Ralstonia solanacearum* race 3 biovar 2 strains	
rk2208.1 F3	GAGAG ACATG TCCGA TTCCG
rk2208.1 B3	GCCGA TGTCA TCAAG CTCAA
rk2208.1 FIP ^1^	TGTGA CTTCC ACGTC AAGCG TTGCA ATCAC CGACT TCCTC A
rk2208.1 BIP ^1^	GCGAG AAGCC CGTGT GCTTG TCACG ATTTT CGGCC AGTT
rk2208.1 loopB	AGAGC TTTTC GCCAA TCGAC T
**Assimilating probes**	
Se F strand loopF ^3^	FAM ^2^—ACGCT GAGGA CCCGG ATGCG AATGC GGATG CGGAT GCCGA **GACGA AAGAG CGTGG TAATT AAC**
Se F strand loopB ^3^	FAM ^2^—ACGCT GAGGA CCCGG ATGCG AATGC GGATG CGGAT GCCGA **GGGCA ATTCG TTATT GGCG**
λ-phage F strand loopF ^3^	TAMRA ^4^—ACGCT GAGGA CCCGG ATGCG AATGC GGATG CGGAT GCCGA **CTGCA TACGA CGTGT CT**
egl62 F strand loopF ^3^	TAMRA ^4^—ACGCT GAGGA CCCGG ATGCG AATGC GGATG CGGAT GCCGA **CCGGG TCATT GATGC CCTT**
rk2208.1 F strand loopB ^3^	FAM ^2^—ACGCT GAGGA CCCGG ATGCG AATGC GGATG CGGAT GCCGA **AGAGC TTTTC GCCAA TCGAC T**
Quench strand	TCGGC ATCCG CATCC GCATT CGCAT CCGGG TCCTC AGCGT—Q ^5^

^1^ Underlined text represents F2/B2 sequence in FIP/BIP primer; ^2^ FAM: 6-carboxyfluorescein; ^3^ Bold text represents loop primer sequence used in Assimilating Probes; ^4^ TAMRA: carboxytetramethylrhodamine; ^5^ Q: Iowa Black Quencher-1, Integrated DNA Technologies, Coralville, IA, USA.

### 3.4. Quantitative LAMP Analysis

For quantitative PCR the amplification efficiency can be estimated from the relationship between threshold cycle and the initial copy number of DNA [43]. To evaluate the speed of LAMP reactions used in these experiments, we use the doubling time (τ_D_) for double stranded DNA assuming exponential amplification [44]. If the observed threshold time (t_T_) corresponds to the generation of a conserved quantity of double stranded DNA Amplicon (A_T_):

(1) AT = A02tTτD

where A_0_ is the initial template DNA quantity. The doubling time then is simply the product of −log(2) and the slope of threshold time *vs.* log(c_i_).

(2) $${\text{t}}_{\text{T}}\;=\;\frac{{\text{τ}}_{\text{D}}}{\log(2)}\left[\log\left(\text{K}\right)-\text{log}({\text{A}}_{0})\right]$$

Detection limits for this work were estimated simplistically as the minimum tested DNA quantity resulting in positive classification (*i.e.*, mathematically determinate t_T_ values based on the available data) for each of a set of triplicate reactions.

## 4. Conclusions

Our results show that spectrally unique Assimilating Probes can be used to develop multiplexed diagnostic reactions using LAMP. Reaction performance (with respect to limit of detection) suffers somewhat (generally by one or two orders of magnitude) when reactions are duplexed due to competitive effects, but our observations indicate that these effects can be largely negated by the use of newer generations of more robust strand-displacing polymerases that are available commercially. These advantages are quite compelling for use in rudimentary labs and in the field as the reactions do not require thermal cycling, and several relatively inexpensive and portable instruments are now available to conduct duplexed, fluorescence based isothermal nucleic acid amplification assays (*i.e.*, Genie III^®^ from Optigene Ltd., Horsham UK; Twista^®^ real-time fluorometer, TwistDx Ltd., Cambridge UK; Smart-DART™ 8-Well V.3, Diagenetix, Inc, Honolulu, HI, USA).

## References

[1] Gill P., Ghaemi A. (2008). Nucleic acid isothermal amplification technologies: A review. Nucleosides Nucleotides Nucleic Acids.

[2] Chang C.C., Chen C.C., Wei S.C., Lu H.H., Liang Y.H., Lin C.W. (2012). Diagnostic devices for isothermal nucleic acid amplification. Sensors.

[3] Jenkins D.M., Kubota R., Dong J., Li Y., Higashiguchi D. (2011). Handheld device for real-time, quantitative, lamp-based detection of *Salmonella enterica* using assimilating probes. Biosens. Bioelectron..

[4] Kubota R., LaBarre P., Singleton J., Beddoe A., Weigl B.H., Alvarez A.M., Jenkins D.M. (2011). Non-instrumented nucleic acid amplification (NINA) for rapid detection of *Ralstonia solanacearum* Race 3 Biovar 2. Biol. Eng. Trans..

[5] Kubota R., LaBarre P., Weigl B.H., Li Y., Haydock P., Jenkins D.M. (2013). Molecular diagnostics in a teacup: Non-Instrumented nucleic acid amplification (NINA) for rapid, low cost detection of *Salmonella enterica*. Chin. Sci. Bull..

[6] Craw P., Balachandran W. (2012). Isothermal nucleic acid amplification technologies for point-of-care diagnostics: A critical review. Lab Chip.

[7] Bissonnette L., Bergeron M.G. (2010). Diagnosing infections—Current and anticipated technologies for point-of-care diagnostics and home-based testing. Clin. Microbiol. Infect..

[8] Bissonnette L., Bergeron M.G. (2012). Infectious disease management through point-of-care personalized medicine molecular diagnostic technologies. J. Pers. Med..

[9] Vincent M., Xu Y., Kong H. (2004). Helicase-dependent isothermal DNA amplification. EMBO Rep..

[10] Piepenburg O., Williams C.H., Stemple D.L., Armes N.A. (2006). DNA detection using recombination proteins. PLoS Biol..

[11] Van Ness J., van Ness L.K., Galas D.J. (2003). Isothermal reactions for the amplification of oligonucleotides. Proc. Natl. Acad. Sci. USA.

[12] Vandervliet G.M.E., Schukkink R.A.F., Vangemen B., Schepers P., Klatser P.R. (1993). Nucleic-acid sequence-based amplification (NASBA) for the identification of mycobacteria. J. Gen. Microbiol..

[13] Notomi T., Okayama H., Masubuchi H., Yonekawa T., Watanabe K., Amino N., Hase T. (2000). Loop-mediated isothermal amplification of DNA. Nucleic Acids Res..

[14] Nagamine K., Watanabe K., Ohtsuka K., Hase T., Notomi T. (2001). Loop-mediated isothermal amplification reaction using a nondenatured template. Clin. Chem..

[15] Nagamine K., Hase T., Notomi T. (2002). Accelerated reaction by loop-mediated isothermal amplification using loop primers. Mol. Cell. Probe.

[16] Kubota K., Jenkins D.M., Alvarez A.M., Su W.W. (2011). Fret-based assimilating probe for sequence-specific real-time monitoring of loop-mediated isothermal amplification (LAMP). Biol. Eng. Trans..

[17] Mori Y., Nagamine K., Tomita N., Notomi T. (2001). Detection of loop-mediated isothermal amplification reaction by turbidity derived from magnesium pyrophosphate formation. Biochem. Biophys. Res. Commun..

[18] Mori Y., Kitao M., Tomita N., Notomi T. (2004). Real-time turbidimetry of LAMP reaction for quantifying template DNA. J. Biochem. Biophys. Methods.

[19] Tomita N., Mori Y., Kanda H., Notomi T. (2008). Loop-mediated isothermal amplification (LAMP) of gene sequences and simple visual detection of products. Nat. Protoc..

[20] Goto M., Honda E., Ogura A., Nomoto A., Hanaki K. (2009). Colorimetric detection of loop-mediated isothermal amplification reaction by using hydroxy naphthol blue. BioTechniques.

[21] Wastling S.L., Picozzi K., Kakembo A.S., Welburn S.C. (2010). LAMP for human African trypanosomiasis: A comparative study of detection formats. PLoS Negl. Trop. Dis..

[22] Corstjens P., Zuiderwijk M., Brink A., Li S., Feindt H., Neidbala R.S., Tanke H. (2001). Use of up-converting phosphor reporters in lateral-flow assays to detect specific nucleic acid sequences: A rapid, sensitive DNA test to identify human papillomavirus type 16 infection. Clin. Chem..

[23] Kiatpathomchai W., Jaroenram W., Arunrut N., Jitrapakdee S., Flegel T.W. (2008). Shrimp Taura syndrome virus detection by reverse transcription loop-mediated isothermal amplification combined with a lateral flow dipstick. J. Virol. Methods.

[24] Tomlinson J.A., Dickinson M.J., Boonham N. (2010). Rapid detection of *Phytophthora ramorum* and *P. Kernoviae* by two-minute DNA extraction followed by isothermal amplification and amplicon detection by generic lateral flow device. Phytopathology.

[25] Iseki H., Alhassan A., Ohta N., Thekisoe O.M., Yokoyama N., Inoue N., Nambota A., Yasuda J., Igarashi I. (2007). Development of a multiplex loop-mediated isothermal amplification (mLAMP) method for the simultaneous detection of bovine babesia parasites. J. Microbiol. Methods.

[26] He L., Xu H.S. (2011). Development of a multiplex loop-mediated isothermal amplification (mLAMP) method for the simultaneous detection of white spot syndrome virus and infectious hypodermal and hematopoietic necrosis virus in penaeid shrimp. Aquaculture.

[27] Shao Y.C., Zhu S.M., Jin C.C., Chen F.S. (2011). Development of multiplex loop-mediated isothermal amplification-RFLP (mLAMP-RFLP) to detect *Salmonella* spp. and *Shigella* spp. in milk. Int. J. Food Microbiol..

[28] Tanner N.A., Zhang Y.H., Evans T.C. (2012). Simultaneous multiple target detection in real-time loop-mediated isothermal amplification. BioTechniques.

[29] Hayward A.C. (1991). Biology and epidemiology of bacterial wilt caused by *Pseudomonas solanacearum*. Annu. Rev. Phytopathol..

[30] Denny T.P. (2006). Plant Pathogenic Ralstonia Species.

[31] Ji P.S., Allen C., Sanchez-Perez A., Yao J., Elphinstone J.G., Jones J.B., Momol A.T. (2007). New diversity of *Ralstonia solanacearum* strains associated with vegetable and ornamental crops in Florida. Plant Dis..

[32] Lambert C.D. (2002). Agricultural bioterrorism protection act of 2002: Possession, use, and transfer of biological; agents and toxins; interim and final rule. 7 CFR Part 331.

[33] Kubota K., Schell M.A., Peckham G.D., Rue J., Alvarez A.M., Allen C. (2011). *In silico* genomic subtraction guides development of highly accurate, DNA-based diagnostics for *Ralstonia solanacearum* Race 3 Biovar 2 and blood disease bacterium. J. Gen. Plant Pathol..

[34] Tanner N.A., Evans T.C. (2014). Loop-mediated isothermal amplification for detection of nucleic acids. Curr. Protoc. Mol. Biol..

[35] Chander Y., Koelbl J., Puckett J., Moser M.J., Klingele A.J., Liles M.R., Carrias A., Mead D.A., Schoenfeld T.W. (2014). A novel thermostable polymerase for RNA and DNA loop-mediated isothermal amplification (LAMP). FMICB.

[36] Markoulatos P., Siafakas N., Moncany M. (2002). Multiplex polymerase chain reaction: A practical approach. J. Clin. Lab. Anal..

[37] Jenkins D.M., Jones J., Kubota R. (2014). Evaluation of portable DNA-based technologies for identification of *Ralstonia solanacearum* Race 3 Biovar 2 in the field. Biol. Eng. Trans..

[38] Norman D., Alvarez A.M. (1989). A rapid method for presumptive identification of *Xanthomonas campestris* pv. *dieffenbachiae* and other Xanthomonads. Plant Dis..

[39] McClelland M., Sanderson K.E., Spieth J., Clifton S.W., Latreille P., Courtney L., Porwollik S., Ali J., Dante M., Du F. (2001). Complete genome sequence of *Salmonella enterica* serovar Typhimurium LT2. Nature.

[40] Salanoubat M., Genin S., Artiguenave F., Gouzy J., Mangenot S., Arlat M., Billault A., Brottier P., Camus J.C., Cattolico L. (2002). Genome sequence of the plant pathogen *Ralstonia solanacearum*. Nature.

[41] Gabriel D.W., Allen C., Schell M., Denny T.P., Greenberg J.T., Duan Y.P., Flores-Cruz Z., Huang Q., Clifford J.M., Presting G. (2006). Identification of open reading frames unique to a select agent: *Ralstonia solanacearum* Race 3 Biovar 2. Mol. Plant Microbe Interact..

[42] Perez A.S., Mejia L., Fegan M., Allen C. (2008). Diversity and distribution of *Ralstonia solanacearum* strains in Guatemala and rare occurrence of tomato fruit infection. Plant Pathol..

[43] Li W.B., Li D.Y., Twieg E., Hartung J.S., Levy L. (2008). Optimized quantification of unculturable *Candidatus* Liberibacter spp. in host plants using real-time PCR. Plant Dis..

[44] Keremane M.L., Ramadugu C., Rodriguez E., Kubota R., Shibata S., Hall D.G., Roose M.L., Jenkins D., Lee R.F. (2015). A rapid field detection system for citrus huanglongbing associated “candidatus liberibacter asiaticus” from the psyllid vector, diaphorina citri kuwayama and its implications in disease management. Crop Prot..

